# Revisiting the discriminatory accuracy of traditional risk factors in preeclampsia screening

**DOI:** 10.1371/journal.pone.0178528

**Published:** 2017-05-25

**Authors:** Merida Rodriguez-Lopez, Philippe Wagner, Raquel Perez-Vicente, Fatima Crispi, Juan Merlo

**Affiliations:** 1 Unit for Social Epidemiology, Faculty of Medicine, Lund University, Malmö, Sweden; 2 Fetal i+D Fetal Medicine Research Center, BCNatal—Barcelona Center for Maternal-Fetal and Neonatal Medicine (Hospital Clínic and Hospital Sant Joan de Deu), Institut Clínic de Ginecologia, Obstetricia i Neonatologia, Institut d'Investigacions Biomèdiques August Pi i Sunyer, Universitat de Barcelona, and Centre for Biomedical Research on Rare Diseases (CIBER-ER), Barcelona, Spain; University of North Carolina at Chapel Hill, UNITED STATES

## Abstract

**Background:**

Preeclampsia (PE) is associated with a high risk of perinatal morbidity and mortality. However, there is no consensus in the definition of high-risk women.

**Aim:**

To question current definition of high PE risk and propose a definition that considers individual heterogeneity to improves risk classification.

**Methods:**

A stratified analysis by parity was conducted using the Swedish Birth Register between 2002–2010 including 626.600 pregnancies. The discriminatory accuracy (DA) of traditional definitions of high-risk women was compared with a new definition based on 1) specific combinations of individual variables and 2) a centile cut-off of the probability of PE predicted by a multiple logistic regression model.

**Results:**

None of the classical risk-factors alone reached an acceptable DA. In multiparous, any combination of a risk-factor with previous PE or HBP reached a +LR>10. The combination of obesity and multiple pregnancy reached a good DA particularly in the presence of previous preeclampsia (positive likelihood ratio (LR+) = 26.5 or chronic hypertension (HBP) LR+ = 40.5. In primiparous, a LR+>15 was observed in multiple pregnancies with the simultaneous presence of obesity and diabetes mellitus or with HBP. Predicted probabilities above 97 centile in multiparous and 99 centile in primiparous provided high (LR+ = 12.5), and moderate (LR+ = 5.85), respectively. No one risk factor alone or in combination provided a LR- sufficiently low to rule-out the disease.

**Conclusions:**

In preeclampsia prediction the combination of specific risk factors provided a better discriminatory accuracy than traditional single risk approach. Our results contribute to a more personalized risk estimation of preeclampsia.

## Introduction

Preeclampsia (PE) is defined as the new onset of hypertension and proteinuria after the 20^th^ gestational week. This complication affects 3–10% of all pregnancies [1], and associates a higher risk of perinatal complication such as intrauterine growth restriction, prematurity and maternal mortality[2]. Although the underlying causes of PE are still unknown[3], a number of different risk-factors have been recognized and are currently recommended in screening guidelines for its early prediction and prevention[4–6]. In fact, low-dose aspirin (LDA) has been proved to reduce the risk of PE in selected high-risk patients and it is usually recommended for preventing PE. However, there is no consensus among guidelines on how to identify those women at higher risk who might benefit from a close follow-up and prophylactic treatment with LDA.

For instance, the *NICE* guideline classifies women as at high-risk for PE when any major risk factor (i.e. previous PE, chronic kidney disease (CKD), autoimmune disease, diabetes mellitus (DM), or chronic hypertension (HBP)) or at least two moderate risk factors (i.e., primiparity, ≥40 years old, inter-pregnancy interval >10 years, obesity at first visit, family history of PE or multiple pregnancy) are present[6]. The *Task Force guidelines*[7], however, defines high-risk for screening when at least one of the above risk factors (major or minor) is present, but recommends LDA only in those with more than one previous PE or one previous PE delivering preterm. The *World Health Organization guideline*[5] define high risk as the existence of at least one major risk factor including multiple pregnancy. Likewise, local guidelines are used in Sweden to define high-risk of PE. For example, in the region of Skåne, Sweden, the definition of high risk includes only major risk factors, and specifies that only those diabetics with vascular damage and those previous PE with severe, early or simultaneous fetal growth restriction should be included[8].

Overall, current screening guidelines are easy to apply in everyday practice as they are not based on complicated risk equations but mainly on the existence of one or two risk factors. The selection of those risk factors is mainly based on the difference in the average risk between exposed and non-exposed women, which is normally appraised by measures of association like the odds ratio (OR). However, this form of screening could be criticized, as previous methodological studies[9–12] have stressed that measures of association alone are inappropriate to discriminate between individuals who will subsequently suffer a disease from those who will not. Actually, what it is often considered as a robust association (e.g., OR≥5), is related to a rather low discriminatory accuracy due to a high false positive fraction (FPF) and/or false negative fraction (FNF) in the population[9–12]. Therefore, current guidelines may translate in an overdiagnosis (thereby unnecessary treatments, avoidable monitoring, and increase parental anxiety) or, on the contrary, underdiagnosis that may prevent a close follow-up of patients at risk of PE.

A suitable approach to improve discriminatory accuracy in clinical decision making could be to adopt the perspective of precision medicine[13] aiming to better understand individual heterogeneity in PE risk and, thereby, achieve a higher predictive accuracy. Therefore, the objective of this study was double. Firstly, using measures of discriminatory accuracy, we aimed to question established risk classification guidelines for PE. Subsequently, we aimed to apply two alternative definitions of high-risk. The first was based on specific combinations of risk factors selected by *random forest analysis*[14] and the second was based on predicted probabilities derived from multiple logistic regression models.

## Methods

### Population and study design

This study is based on the Swedish Medical Birth Registry (MBR) that includes detailed and standardized information on nearly all pregnancies in Sweden culminating in delivery. Overall, the quality of the register is rather high as described elsewhere[15,16]. Using the unique Swedish personal identification number, the National Board of Health and Welfare and Statistics in Sweden linked the MBR to the Patient register that records all inpatient and outpatient hospital diagnoses. In addition, the longitudinal integration database for health insurance and labour market studies (LISA) records information on socioeconomic factors. Data is codified by a personal identification code without access to the real personal identification number in order to protect the participants’ anonymity. The regional ethical review board in southern Sweden approved the database use and did not required the explicit informed consent from women.

**Fig 1:**
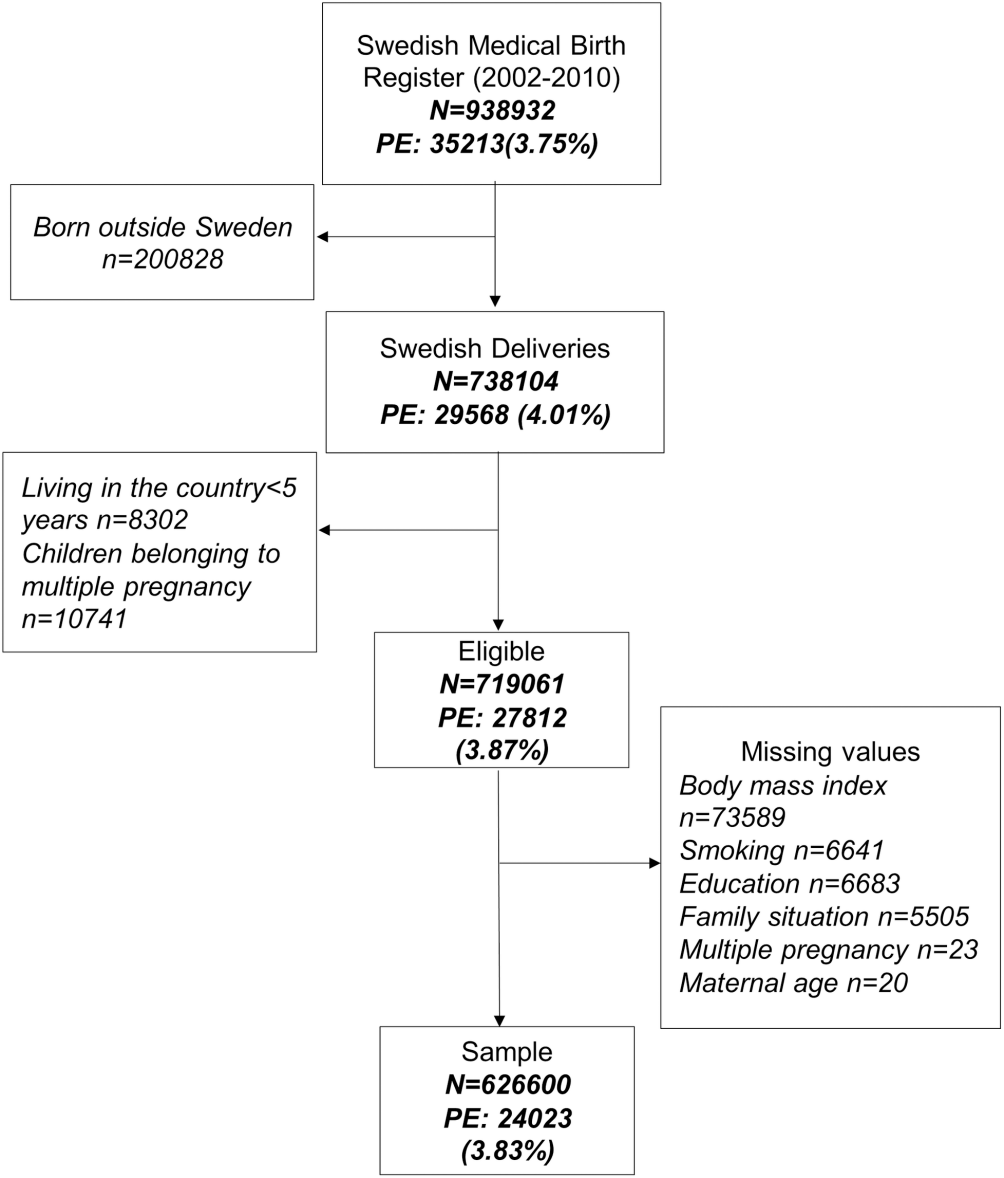
Flow diagram showing the selection of study population of deliveries in the Swedish birth register.

We identified all the 938.932 deliveries recorded in the MBR from 1^st^ January 2002 to 31^th^ December 2010. We decided to restrict the analysis to deliveries from Swedish mothers that has been residing in Sweden for more or equal to 5 years in order to improve the homogeneity, completeness and validity of the information. To prevent that the same episode of PE counted twice in multiple pregnancies, only one children were randomly selected among those belonging to the same pregnancy. The flow diagram of the study population is shown in Fig 1. The final study sample consisted of 626.600 pregnancies, representing 67% of the initial population. The prevalence of PE was close to 4% in both the final sample and the original population. Characteristics of the included and excluded population is shown in supplemental data (S1 Table).

### Assessment of variables

The main outcome of our study was PE or eclampsia defined as a diagnosis coded O14 or O15, respectively, according the International Classification of Diseases 10^th^ version (ICD-10). In this manuscript both pathologies are named PE. PE was recoded as positive whether it was present in the MBR or in the Patient register from 20 weeks of gestational age and up to six weeks after delivery. Gestational hypertension (ICD-10 code O13), chronic hypertension with proteinuria (O11) and edema and proteinuria (O12) were included as non-PE. Non-specified hypertensive disorder (O16) was considered as HBP. When the same patient was categorized as Gestational hypertension and PE was included as PE as previous studies with the MBR[17].

All the exposure variables were dichotomized in order to follow a similar approach as in current clinical guidelines and categorized as: maternal age into <40 or ≥40 years, educational achievement as ≤11 years or ≥12 years of formal education obtained the year before the delivery and family situation as cohabiting with the child’s father or not. The presence of disease (ICD-10 codes) was identified at the first antenatal visit, at the hospital discharge after delivery and/or during a five-year period before pregnancy, and was considered as positive whether it was present in the MBR or in the Patient register. The codes obtained were: HBP (I10-I15)), DM (E10, E11-E14)), CKD (N18, N19)), and autoimmune diseases including systemic lupus erythematosus (M32) and rheumatoid arthritis (M05, M06). There were only 26 pregnancies with antiphospholipid syndrome (D686), therefore it was included together with autoimmune diseases. Obesity was defined as body mass index (BMI) ≥ 30kg/m^2^[17–19]. Information on smoking habits before pregnancy was self-reported and concerned the status up to 3 months before pregnancy, and dichotomized into smoker (including mild: 1–9 and heavy ≥10 cigarettes per day) or non-smoker. Previous PE was defined whether O14-O15 codes were present in the Patient register from 270 days and up to five-year period before birthdate. Parity was dichotomized into primiparous or multiparous. Pregnancy characteristics included multiple pregnancy, conception by assisted reproductive technologies (ART) and gestational diabetes (O24).

In the MBR, the data concerning the current pregnancy is collected prospectively, before onset of potential adverse pregnancy outcomes, which prevents recall bias. PE diagnose is noted by the responsible doctor at discharge from hospital. The information about chronic hypertension and other maternal demographic, clinical and reproductive characteristics are recorded by a midwife during mother´s first visit for antenatal care. Then, the data is forwarded to the MBR where the information is computerized. The records are standardized and are identical throughout the country, which minimizes information bias.

### Statistical analysis

#### Stratified analysis by parity

To decide whether to perform the analysis in the overall population or stratified by parity, we explored the interaction effect between each factor and parity by adding one interaction term to the baseline logistic model. Each model was specified as i = 1 if nulliparous and 0 if multiparous, and j = 1 when the risk factor was present and 0 otherwise. Then, OR11, OR10 and OR01 was obtained, OR00 was considered the reference category. Multiplicative interaction was determined by the ratio of Odds ratio (ratio of ORs = OR11 / (OR10 x OR01), directly obtained from the output of multiple logistic models. Additive interaction was determined by the relative excess of risk due to interaction relative to the risk without exposure also called Interaction contrast ratio (ICR = OR11−OR10−OR01+1), using the regression coefficients and covariance matrix obtained from the multiple logistic regressions. When the confidence interval did not include the value of one or zero, the interaction was considered statistically significant in the multiplicative or additive scale, respectively. The interaction was classified as positive in the multiplicative/additive scale if ratio of ORs>1/ ICR>0, negative if ratio if ORs<1/ICR<0 or absent if ratio of ORs = 1/ICR = 0. As significant modification was found in both multiplicative and the additive scales, all analyses were stratified on parity, also considering that previous PE can only be present in multiparous woman.

The prevalence of risk factors in women with and without PE was calculated using point estimations and confidence intervals. Logistic regression analysis was performed to obtain crude and adjusted ORs stratified by parity. Starting from a saturated model containing all the variables, a backward elimination strategy was applied to construct the final multiple regression model. A significance level of 0.1 was defined to exclude variables from the saturated model. When the confidence interval did not contain the null, the difference was considered statistically significant. Since the data was correlated (children from different pregnancies clustered within mothers) we obtained robust standard errors and 95%CI. Models fit were compared by Akaike information criteria (AIC). Thereafter, a variable based on the number of current risk factors[4–6] (i.e. previous PE, CKD, autoimmune disease, DM, HBP, ≥40 years old, obesity, multiple pregnancy and including ART and gestational diabetes) was created and then we explored whether their combinations could increase the risk of PE by subgroups of parity. Smoking, cohabiting and education achievement were included as covariates in multiple logistic regression models.

#### Current and new definition of high-risk groups for PE

To identify women at the highest PE risk, we first adopted a similar approach as that used by current guidelines[4–6], and recreated three different classification of high risk of PE as having 1) one risk factor, 2) one major or two moderate and 3) one major including multiple pregnancy as explained in the introduction section. Information on family history of PE and pregnancy intervals was not available in all women. We included instead gestational diabetes and conception by ART as moderate risk factors. Then, random forest (RF)[14] was performed to guide variable selection for specific risk combination. The RF analysis also included smoking habits, cohabiting and education achievement. In short, RF fits many classification trees by randomly selected predictors and creating different trees by bootstrapping techniques. For this reason, RF produces more stable and accurate predictions than a single tree analysis. This algorithm allowed to identify the most important variables for splitting the data and therefore were subsequently used to create subgroups. RF are covered in more detail elsewhere[14–20]. Finally, we also created subgroups of risk based on predicted probabilities derived from multiple logistic regression model. For this purpose, the predicted probabilities were dichotomized using three cut-offs and those women (> 95, >97 and >99 centiles) were considered as higher risk.

#### Discriminatory accuracy of high risk groups

The discriminatory accuracy of the high-risk definition was determined based on a 1) single risk factors, 2) recreating three guidelines approach described above[5–7], 3) subgroups generated using RF and 4) individual risk probabilities predicted by multiple regression analysis. As previous PE and HBP are usually well recognized by clinicians as very important risk factors for PE, we additionally evaluate the performance of the selected combination of risk factors among those without these conditions. For the same reason, an additional analysis was performed to identify combinations of risk factors associated with a higher risk of PE among those without major factors.

The absolute risk (AR), attributable risk (AF), true positive fraction (TPF), false positive fraction (FPF), and likelihood ratio (LR) were calculated. Briefly, the LR shows the probability of having or not the risk factor (i.e., positive or negative) in patients with PE and compare to the probability of the same results in patients without PE. The null value is 1. In general, a LR+ >10 is considered high enough to rule-in PE, 5–10 moderate and 2–5 small[21,22]. The established criteria to rule-in PE is a LR+ > 10 and LR- < 0.2 to rule-out the disease with confidence[7]. We also evaluated the area under the ROC curve (AUC) and TPF for a FPF of 10% of the multiple logistic regression models. To consider the assumption of independency for RF, we chose one birth randomly from each multiparous mother. This strategy was also applied to obtained the measures of DA but the results were similar than those obtained keeping the clustered data. Therefore, we reported all results keeping the clustered data in multiparous. Analyses were performed using STATA 14 (Statacorp, College Station, Texas, US) and the randomForest Package in R version 0.99.893 (R Foundation for Statistical Computing, Vienna, Austria).

## Results

### Heterogeneity of risk factors for preeclampsia and parity

Preeclampsia was present in 3.83% of the population. The characteristics of the deliveries in multiparous and primiparous are presented in Table 1. Primiparous women presented a higher incidence of PE, lower education, and higher rate of no cohabiting with children’s father, smoking before pregnancy and conception by ART when compared to multiparous. The rest of the risk factors were higher in multiparous group with the exception of autoimmune diseases that showed the same prevalence in both groups. In the overall population, DA of primiparity in relation to PE was rather low with a LR+ of 1.48 (1.46–1.49) and a LR- of 0.62 (0.61–0.63).

**Table 1:** Sociodemographic, pre-pregnancy and pregnancy characteristics of the 626600 Swedish deliveries recorded between 2002 and 2010 stratified by parity.

	MultiparousN = 344001	PrimiparousN = 282599
Preeclampsia	8291 (2.41)	15732 (5.57)*
***Sociodemographic***		
Maternal age		
<40	328384 (95.46)	278016 (98.38)
≥40	15617 (4.54)	4583 (1.62)*
Educational status		
Medium-High	136942 (39.81)	125662 (44.47)
Low	207059 (60.19)	156937 (55.53)*
Family situation		
Cohabiting	33148 (97.14)	265590 (93.98)
Non-Cohabiting	9853 (2.86)	17009 (6.02)*
***Pre-pregnancy***		
DM		
No	341490 (99.27)	280899 (99.40)
Yes	2511 (0.73)	1700 (0.60)*
HBP		
No	341150 (99.17)	280523 (99.27)
Yes	2851 (0.83)	2076 (0.73)*
Autoinmune disorders		
No	343230 (99.78)	281984 (99.78)
Yes	771 (0.22)	615 (0.22)
CKD		
No	342153 (99.456)	281266 (99.53)
Yes	1848 (0.54)	1333 (0.47)*
Obesity		
No	299824 (87.16)	254513 (90.06)
Yes	44177 (12.84)	28086 (9.94)*
Previous preeclampsia		
No	330594 (96.10)	NA
Yes	13407 (3.90)	
Smoker		
No	292038 (84.89)	223392 (79.05)
Yes	51963 (15.11)	59207 (20.95)*
***Pregnancy***		
Multiple pregnancy		
No	338376 (98.36)	279145 (98.78)
Yes	5625 (1.64)	3454 (1.22)*
Conception by ART		
No	338838 (98.50)	271486 (96.07)
Yes	5163 (1.50)	11113 (3.93)*
Gestational diabetes		
No	341039 (99.14)	280536 (99.27)
Yes	2962 (0.86)	2063 (0.73)*

Data are N (%).

*p value <0.001 from cluster-weighted *Χ*^2^ test

ART: assisted reproductive technology, CKD: Chronic kidney disease, DM: Diabetes Mellitus, HBP: chronic hypertension NA: Non applicable.

**Fig 2:**
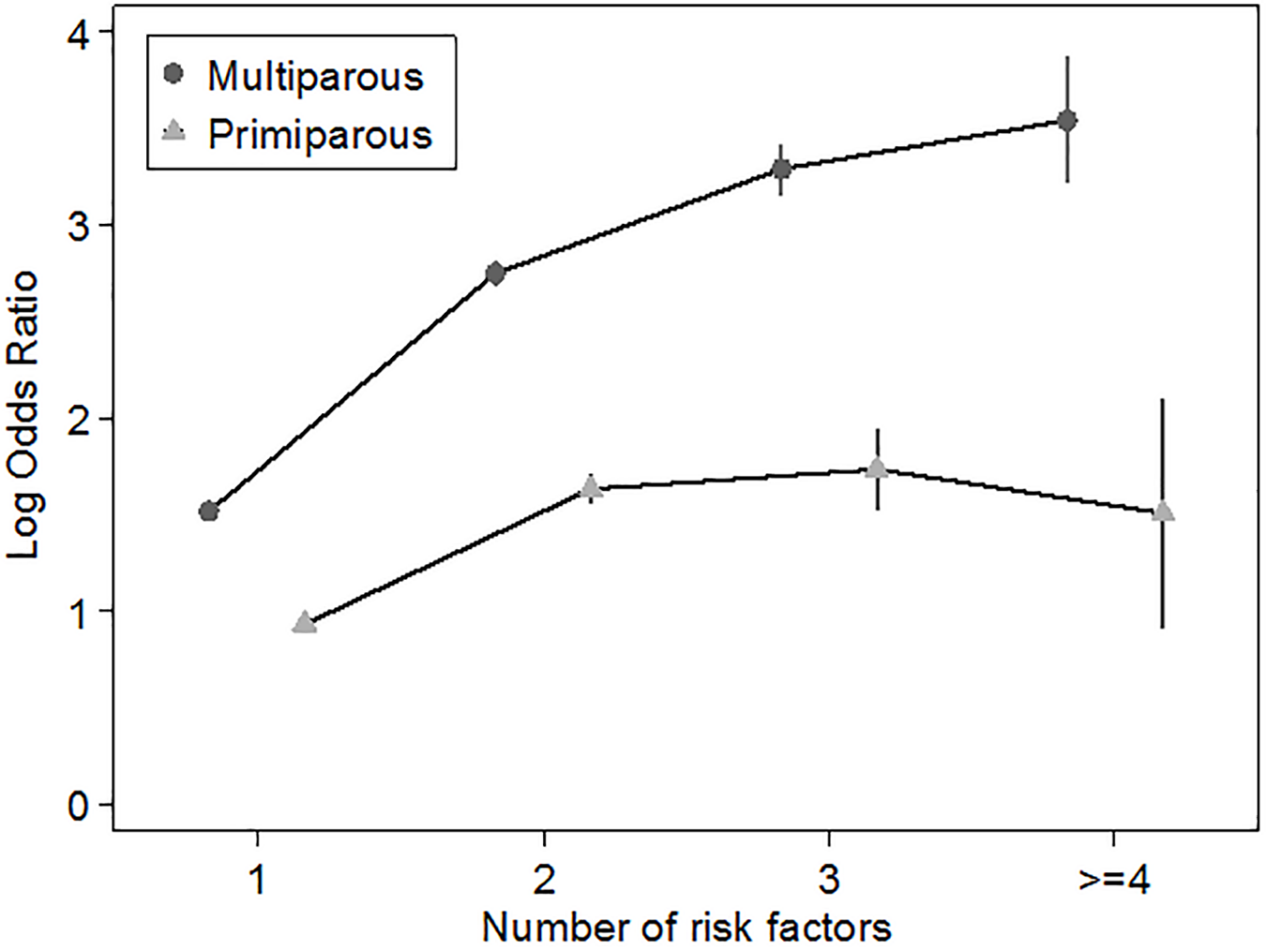
Log-odds ratio for preeclampsia according to the number of clinical risk factors in multiparous and primiparous women adjusted by smoking, education and family situation.

An interaction effect was observed between each traditional risk factors and parity, in one or both scales. The effect of risk factors on PE was lower in nulliparous than in multiparous. The lowest ICR (ICR<2) was observed for the interaction of parity with HBP and with multiple pregnancy. An opposite direction of the interaction measures was observed between parity and diabetes and between parity and smoking.(S2 Table). 35.45% of PE cases occurred in pregnancies without a known risk factor in multiparous and in 64.75% among primiparous. Tables 2 and 3 show the relationship between PE and sociodemographic, pre-pregnancy and pregnancy characteristics among multiparous and primiparous women, respectively. In multiparous, all variables were positively associated with PE, with particularly strong associations for HBP, previous PE and multiple pregnancy. In both groups, the risk of PE was lower in smokers, while autoimmune disease was no longer associated with PE after adjustment. Despite the heterogeneity of the effect by parity, the direction of associations observed in multiple model was similar but family situation remained significant only among multiparous. The adjusted OR associated with unspecific combination of risk factors was greater in multiparous than in primiparous, even when the analysis was restricted to those mothers without previous PE (S3 Table). For illustrative purposes, Fig 2 shows the magnitude of OR (in logarithm scale) with the increment of the number of clinical risk factors stratified by parity.

**Table 2:** Relation between sociodemographic, pre-pregnancy and pregnancy characteristics and preeclampsia (PE) in the 344001 Swedish deliveries from multiparous woman recorded between 2002 and 2010.

Risk Factor	AR	AF	TPF (95% CI)	FPF (95% CI)	LR+ (95% IC)	LR- (95% CI)	ORc (95% CI)	ORa (95% CI)
Maternal age, y								
<40	2.33						Ref	Ref
≥40	4.04	1.71	7.6 (7–8.2)	4.5 (4.4–4.5)	1.70 (1.58–1.84)	0.97 (0.96–0.97)	1.76 (1.62–1.91)	1.73 (1.58–1.89)
Educational status								
Medium-High	2						Ref	Ref
Low	2.68	0.68	66.9 (65.9–67.9)	60 (59.2–60.2)	1.12 (1.10–1.13)	0.83 (0.80–0.85)	1.35 (1.29–1.41)	1.20 (1.14–1.26)
Family situation								
Cohabiting	2.39						Ref	Ref
Non-Cohabiting	3.04	0.65	3.6 (3.2–4)	2.8 (2.8–2.9)	1.27 (1.14–1.42)	0.99 (0.99–1)	1.28 (1.14–1.44)	1.24 (1.09–1.41)
Diabetes								
No	2.35						Ref	Ref
Yes	10.5	8.16	3.2 (2.8–3.6)	0.7 (0.6–0.7)	4.76 (4.20–5.39)	0.97 (0.97–0.98)	4.88 (4.26–5.57)	2.64 (2.25–3.10)
HBP								
No	2.26						Ref	Ref
Yes	20.1	17.8	6.9 (6.46–7.5)	0.7 (0.7–0.7)	10.1 (9.30–11.1)	0.94 (0.93–0.94)	10.8 (9.85–11.9)	4.63 (4.07–5.27)
Autoinmune								
No	2.40						Ref	Ref
Yes	5.58	3.18	0.5 (0.4–0.7)	0.2 (0.2–0.2)	2.39 (1.76–3.25)	1	2.39 (1.74–3.31)	1.54 (1.06–2.24)
CKD								
No	2.38						Ref	Ref
Yes	7.52	5.14	1.7(1.4–2)	0.1 (0.1–0.1)	3.29 (2.77–3.91)	0.99 (0.99–0.99)	3.33 (2.78–3.99)	2.15 (1.73–2.68)
Obesity								
No	1.91						Ref	Ref
Yes	5.80	3.89	30.9 (29.9–31.9)	12.4 (12.3–12.5)	2.49 (2.41–2.58)	0.79 (0.78–0.80)	3.16 (3.01–3.32)	2.44 (2.31–2.58)
Previous PE								
No	1.76						Ref	Ref
Yes	18.4	16.6	19.7 (28.7–30.7)	3.3 (3.2–3.3)	9.10 (8.76–9.45)	0.73 (0.72–0.74)	12.51 (11.9–13.2)	10.6 (10–11.25)
Smoker								
No	2.43						Ref	Ref
Yes	2.30	-0.13	14.4 (13.7–15.2)	15.1(15–15.2)	0.95 (0.91–1.01)	1.01 (1–1.02)	0.95 (0.89–1)	0.90 (0.84–0.97)
Multiple pregnancy								
No	2.28						Ref	Ref
Yes	10.1	7.82	6.9 (6.3–7.4)	1.5 (1.5–1.5)	4.55 (4.18–4.95)	0.95 (0.94–0.95)	4.88 (4.40–5.26)	5.42 (4.91–5.99)
Conception by ART								
No	2.37						Ref	Ref
Yes	5	2.63	3.1 (2.7–3.5)	1.5 (1.4–1.5)	2.13 (1.88–2.41)	0.98 (0.98–0.99)	2.16 (1.90–2.46)	1.71 (1.48–1.96)
Gestational diabetes								
No	2.37						Ref	Ref
Yes	7.49	5.12	2.7 (2.3–3)	0.8 (0.8–0.8)	3.28 (2.87–3.75)	0.98 (0.98–0.98)	3.34 (2.91–3.84)	1.85 (1.57–2.19)

AF: Attributable fraction, AR: Attributable risk, TPF: True positive fraction, ORc: crude Odds ratio, ORa: mutually adjusted Odds ratios, LR+: Positive Likelihood ratio, LR-: Negative Likelihood ratio, ART: assisted reproductive technology, CKD: Chronic kidney disease.

**Table 3:** Relation between sociodemographic, pre-pregnancy and pregnancy characteristics and risk of preeclampsia (PE) in the 282599 Swedish deliveries from nulliparous woman recorded between 2002 and 2010.

Risk Factor	AR	AF	TPF (95% CI)	FPF (95% CI)	LR+ (95% IC)	LR- (95% CI)	Orc (95% CI)	ORa (95% CI)
Maternal age, y								
<40	5.51						Ref	Ref
≥40	9.14	3.63	2.7 (2.4–2.9)	0.6 (0.5–0.6)	1.71 (1.55–1.88)	0.99 (0.99–0.99)	1.73 (1.56–1.91)	1.49 (1.31–1.62)
Educational status								
Medium-High	5.06						Ref	Ref
Low	5.97	0.91	59.6 (58.8–60.3)	55.3 (55.1–55.5)	1.08 (1.06–1.09)	0.90 (0.89–0.92)	1.19 (1.15–1.23)	1.13 (1.09–1.17)
Family situation								
Cohabiting	5.58						Ref	Ref
Non-Cohabiting	5.36	-0.22	5.8 (5.4–6.2)	6 (5.9–6.1)	0.96 (0.90–1.02)	1	0.96 (0.89–1.02)	0.94 (0.88–1.01)
Diabetes								
No	5.46						Ref	Ref
Yes	22.7	17.3	2.5 (2.2–2.7)	0.5 (0.5–0.5)	4.98 (4.45–5.58)	0.98 (0.98–0.98)	5.08 (4.53–5.70)	4.29 (3.77–4.88)
HBP								
No	5.53						Ref	Ref
Yes	24.1	18.5	3.2 (2.9–3.5)	0.6 (0.6–0.6)	5.37 (4.86–5.93)	0.97 (0.97–0.98)	5.51 (4.98–6.10)	4.07 (3.63–4.57)
Autoimmune								
No	5.56						Ref	Ref
Yes	9.27	3.71	0.4 (0.3–0.5)	0.2 (0.2–0.2)	1.73 (1.32–2.27)	1	1.73 (1.32–2.28)	1.12 (0.78–1.60)
CKD								
No	5.55						Ref	Ref
Yes	10.1	4.50	0.9 (0.7–1)	0.4 (0.4–0.5)	1.90 (1.59–2.26)	1 (0.99–1)	1.90 (1.59–2.28)	1.34 (1.09–1.66)
Obesity								
No	4.84						Ref	Ref
Yes	12.1	7.28	21.6 (21–22.3)	9.2 (9.1–9.4)	2.34 (2.27–2.42)	0.86 (0.86–0.87)	2.71 (2.60–2.82)	2.56 (2.45–2.66)
Smoker								
No	5.68						Ref	Ref
Yes	5.13	-0.55	19.3 (18.7–19.9)	21 (20.9–21.2)	0.92 (0.89–0.95)	1.02 (1.01–1.03)	0.89 (0.86–0.93)	0.83 (0.79–0.88)
Multiple pregnancy								
No	5.39						Ref	Ref
Yes	19.7	14.3	4.3 (4–4.7)	1 (1–1.1)	4.17 (3.84–4.52)	0.97 (0.96–0.97)	4.31 (3.96–4.69)	4.27 (3.90–4.67)
Conception by ART								
No	5.48						Ref	Ref
Yes	7.59	2.11	5.4 (5–5.7)	3.8 (3.8–3.9)	1.39 (1.30–1.49)	0.98 (0.98–0.99)	1.41 (1.32–1.52)	1.17 (1.08–1.25)
Gestational diabetes								
No	5.51						Ref	Ref
Yes	13.9	8.40	1.8 (1.6–2)	0.7 (0.6–0.7)	2.74 (2.42–3.10)	0.99 (0.99–0.99)	2.77 (2.44–3.14)	2.04 (1.79–2.33)

AF: Attributable fraction, AR: Attributable risk, TPF: True positive fraction, ORc: crude Odds ratio; ORa: mutually adjusted Odds ratios, LR+: Positive Likelihood ratio, LR-: Negative Likelihood ratio, ART: assisted reproductive technology, CKD: Chronic kidney disease, HBP: Chronic hypertension.

### Accuracy of current definition of high- risk groups

Concerning a single factor, a LR+ around 10 was only present in multiparous women with HBP or previous PE. No one of the studied variables showed a LR- lower than 0.2 (Tables 2 and 3). Table 4 shows the DA of the high-risk definition analogous to the current guidelines approach. The higher sensitivity was observed when at least one risk factor was present. The inclusion of multiple pregnancy or two moderate risk factors reached similar DA. In the overall population without a major risk factor no one combination of moderate risk-factors reached a LR≥10 neither a LR≤0.2 (S4 Table). The combination of primiparity and multiple pregnancy showed the higher OR, DA (LR+ = 6.66) and absolute risk or positive predictive value (AR = 18.61%).

**Table 4:** Discriminatory accuracy of the traditional approach for defining high- risk for preeclampsia in multiparous and in primiparous women.

Criteria	N	AR	AF	TPF (95% CI)	FPF (95% CI)	LR+ (95% CI)	LR- (95% CI)	ORa (95% CI)*	AUC (95% CI) AIC*
***Multiparous***									
Any Risk	81162	6.59	5.47	64.6 (63.5–65.6)	22.6 (22.4–22.7)	2.86 (2.81–2.91)	0.46 (0.44–0.47)	6.18 (5.90–6.47)	72.0 (71.5–72.6) 71652
1 major or ≥2 moderate	25135	13.8	12.3	41.9 (40.9–43)	6.5 (6.4–6.5)	6.50 (6.35–6.69)	0.62 (0.61–0.63)	10.3 (9.87–10.8)	69.9 (69–70.6) 70031
1major and Multiple pregnancy	25218	13.9	12.5	42.5 (41.4–43.5)	6.5 (6.4–6.5)	6.57 (6.39–6.76)	0.62 (0.60–0.63)	10.6 (10.1–11.1)	70.6 (69–71.2) 69805
***Primiparous***									
Any Risk	48924	11.3	6.97	35.2 (34.5–36)	16.3 (16.1–16.4)	2.17 (2.12–2.22)	0.77 (0.76–0.78)	2.77 (2.69–2.87)	60.9 (60.4–61.4) 118237
1 major or ≥2 moderate	9235	17.9	12.7	10.5 (10–11)	2.8 (2.9–2.8)	3.69 (3.51–3.88)	0.92 (0.92–0.93)	3.96 (3.75–4.19)	56.2 (55.8–56.7) 119488
1major and Multiple pregnancy	8769	19.1	14.1	10.6 (10.2–11.1)	2.7 (2.6–2.7)	4 (3.80–4.21)	0.92 (0.91–0.92)	4.35 (4.11–4.60)	56.5 (56.3–57) 119310

Major risk factors included previous preeclampsia, chronic kidney disease, autoimmune disease, diabetes mellitus or chronic hypertension. Moderate risk factors included, maternal age≥40 years old, obesity at first visit, multiple pregnancy; conception by assisted reproductive technologies and multiple pregnancy.

*data obtained from multiple regression model adjusted by smoke, educational status and family situation AIC: Akaike information criteria, AF: attributable fraction, AR: attributable risk, TPF: true positive fraction, ORa: adjusted Odds ratios by smoker, educational status and family situation. LR+: Positive Likelihood ratio; LR-: Negative Likelihood ratio, CI: confidence interval.

### Accuracy of an alternative definition of high risk for preeclampsia in multiparous vs primiparous

**Fig 3:**
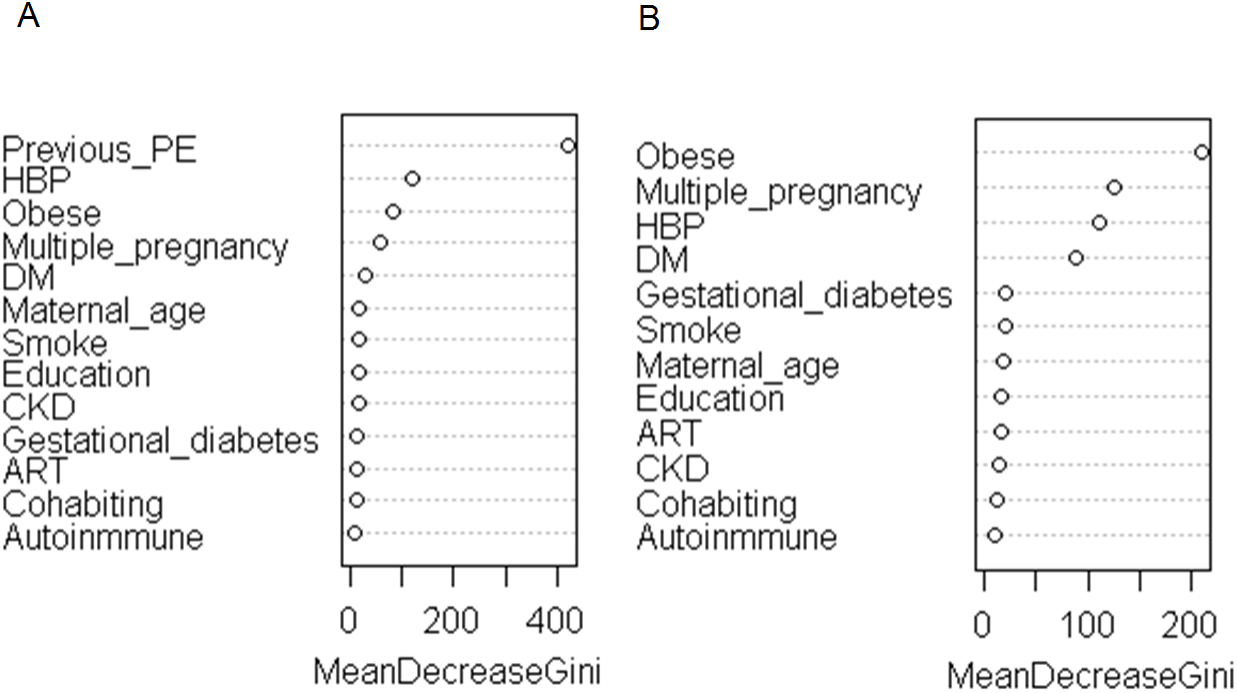
Criteria for risk factor combination based on variable importance in A) Multiparous B) Primiparous for the prediction of preeclampsia according to Random Forest.

Fig 3 shows the most relevant variables for classifying women in relation to their PE risk according the results from the random forest analyses. Previous PE was the most relevant variable among multiparous while obesity was the most relevant variable in primiparous. In multiparous, a combination of multiple pregnancy and obesity with HBP or previous PE was related to the greatest PE risk with an OR above 50 (Table 5). Any combination of a risk factor with previous PE or HBP also presented a LR+>10 (data no shown). In those without previous PE nor HBP, a LR>10 was also observed when multiple pregnancy and obesity were simultaneously combined with: DM (n = 9, LR+ = 18(3.5–81.1), ERC (n = 4, LR+ = 19.7(2.05–189), ART (n = 59, LR+ = 30.3(17.6–51.9) or >40 years (n = 41, LR+ = 12.2(5.39–27.4)). Among those without major risk factors, we also observed that only specific combinations of risk factors were associated with a greater risk of PE (S5 Table). Among those that were spontaneously conceived without major risk factors, multiple pregnancy and gestational diabetes was also associated with a higher risk in non-obese mothers (n = 22, LR+18.30(6.75–49).

**Table 5:** Discriminatory accuracy for specific combinations of risk factor for preeclampsia based on random forest variable importance.

	Combination			n	AR	AF	TPF (95% CI)	FPF (95% CI)	LR+ (95% CI)	LR- (95% CI)	ORa (95% CI)
***Multiparous Incidence PE 2*.*41%***											
PP	HBP	Obese	Multiple	283462	1.23	Ref					
0	0	0	1	4698	8.56	7.33	4.8 (4.4–5)	1.3 (1.2–1.3)	3.79 (3.43–4.19)	0.96 (0.96–0.97)	7.02 (6.28–7.83)
0	0	1	0	39533	3.79	2.56	18.1 (17.3–18.9)	11.3 (11.2–11.4)	1.60 (1.25–1.62)	0.92 (0.91–0.93)	3.05 (2.86–3.25)
0	0	1	1	708	13	11.8	1.1 (0.9–1.4)	0.2 (0.2–0.2)	6.05 (4.86–7.52)	0.99 (0.99–0.99)	11.1 (8.86–13.8)
0	1	0	0	1453	14.7	13.5	2.6 (2.2–2.9)	0.4 (0.3–0.4)	6.96 (6.02–8.03)	0.98 (0.97–0.98)	11.6 (9.94–13.6)
0	1	0	1	24	16.7	15.5	~0	~0	8.10 (2.77–23.7)	~1	14.6 (4.86–43.9)
0	1	1	0	706	19.6	18.4	1.7 (1.4–2)	0.2 (0.1–0.2)	9.84 (8.18–11.8)	0.99 (0.98–0.99)	16.2 (13.2–19.8)
0	1	1	1	10	50	48.8	0.1 (0–0.1)	~0	**40.5 (11.7–139)**	~1	57.2 (9.99–328)
1	0	0	0	9635	15.1	13.9	17.5 (16.7–18.4)	2.4 (2.4–2.5)	7.20 (6.84–7.58)	0.85 (0.84–0.85)	14 (13.2–15.2)
1	0	0	1	136	34.6	33.4	0.6 (0.4–0.6)	~0	**21.4 (15–30.4)**	0.99 (0.99–1)	41 (28.7–58.8)
1	0	1	0	2935	24.8	23.6	8.8 (8.2–9.4)	0.3 (0.2–0.3)	**13.4 (12.3–14.5)**	0.92 (0.91–0.92)	25.8 (23.5–28.3)
1	0	1	1	43	39.5	38.3	0.2 (0.1–0.3)	~0	**26.5 (14.4–48.8)**	~1	51.9 (28–96.5)
1	1	0	0	410	31.5	30.3	1.6 (1.3–1.8)	0.1 (0.1–0.1)	**18.6 (11.6–22.9)**	0.99 (0.98–0.99)	32 (26.2–40.6)
1	1	0	1	6	16.7	15.5	~0	~0	8.10 (0.95–69.3)	~1	13.9 (1.8–105)
1	1	1	0	242	33.8	32.6	1 (0.8–1.2)	~0	**20.8 (15.9–27.1)**	0.99 (0.99–0.99)	36.1 (27.6–40.6)
Multiple model including single variables							AUC:75.35(74.74–75.97) Sensitivity (FP10%):45.01(43.87–46.15) AIC:67934				
Multiple model including combination of variables							AUC:75.23(74.61–75.85) Sensitivity (FP10%):43.34(42.23–44.44) AIC:67632				
***Primiparous Incidence PE = 5*.*57%***											
DM	HBP	Obese	Multiple	248600	4.47	Ref					
0	0	0	1	3056	19.1	14.6	3.7 (3.4–4)	0.9 (0.9–1)	4 (3.66–4.37)	0.97 (0.97–0.97)	4.84 (4.14–5.31)
0	0	1	0	26939	11.5	7.03	19.6 (19–20.2)	8.9 (8.8–9)	2.19 (2.12–2.27)	0.88 (0.88–0.89)	2.69 (2.59–2.81)
0	0	1	1	358	23.5	19	0.5 (0.4–0.5)	0.1 (0.1–0.1)	5.20 (4.07–6.64)	1 (0.99–1)	6.10 (4.76–7.84)
0	1	0	0	1418	22.5	18	2 (1.8–2.2)	0.4 (0.4–0.4)	4.88 (4.31–5.53)	0.98 (0.98–0.99)	5.87 (5.16–6.67)
0	1	0	1	17	47.1	42.6	0.1 (0-.01)	~0	**15.1 (5.82–39)**	~1	17.4 (6.63–45.5)
0	1	1	0	505	29.7	25.2	1 (0.8–1.1)	0.1 (0.1–0.11)	**7.17 (5.93–8.67)**	0.99 (0.99–0.99)	8.15 (6.70–9.91)
0	1	1	1	6	16.7	12.2	~0	~0	3.39 (0.40–29)	~1	3.74 (0.38–36.4)
1	0	0	0	1316	21.7	17.2	1.8 (1.6–2)	0.4 (0.4–0.4)	4.71 (4.14–5.36)	0.99 (0.99–0.99)	5.91 (5.18–6.76)
1	0	0	1	13	23.1	18.6	~0	~0	5.09 (1.40–18.5)	~1	6.03 (1.60–22.6)
1	0	1	0	237	30.4	25.9	0.5 (0.4–0.6)	0.1 (0.1–0.1)	**7.40 (5.61–9.76)**	~1	8.99 (6.80–11.9)
1	0	1	1	4	50	45.5	~0	~0	**16.96 (2.39–120)**	~1	18.9 (2.98–120)
1	1	0	0	93	12.9	8.43	0.1 (0–0.1)	~0	2.51 (1.37–4.61)	~1	2.42 (1.24–4.69)
1	1	1	0	37	29.7	25.2	0.1 (0–0.1)	~0	**7.18 (3.55–14.5)**	~1	7.84 (3.84–16)
Multiple model including single variables							AUC: 61.48(60.99–61.96) Sensitivity (FP10%): 24.73(24.04–25.44) AIC:117232				
Multiple model including combination of variables							AUC: 61.50(61.02–61.98) Sensitivity (FP10%): 24.72(24.05–25.39) AIC:117060				

AIC: Akaike information criteria, AF: Attributable fraction, AR: Attributable risk, TPF: True positive fraction, ORa: mutually adjusted Odds ratios. LR+: Positive Likelihood ratio; LR-: Negative Likelihood ratio, HBP: chronic hypertension; DM: Diabetes Mellitus

Regarding primiparous, the combination of multiple pregnancy and obesity increased the risk particularly in the presence of DM. The combination of HBP and multiple pregnancy also presented a LR>10. A moderate LR was observed in those with HBP and obesity, DM and obesity and DM with multiple pregnancy. There were no patients with these 4 factors neither with the combination of DM, HBP and multiple pregnancy. In the absence of HBP, the combination of multiple pregnancy and obesity with gestational diabetes reached a moderate LR+ (n = 9, LR+ = 8.71(2.18–34.8) but a predictive value of 33%. In primiparous with no major risk factors, no one combination reached a LR>10 but a moderate LR was observed when multiple pregnancy was present in mother older than 40 or with obesity.

The AUC and sensitivity for the model including all variables was higher in multiparous when compared to primiparous(Table 5). Based on multiple regression model, those with a predicted probability>97 centile showed a LR>10 in multiparous (Table 6). In primiparous, those with a predicted probability >99centile showed a moderate LR+. The absence of any single risk factor, neither specific combination nor lower centiles from multiple regression model, reached a LR-below 0.2 in multiparous and primiparous. A summary of the highest risk group is shown in Table 7.

**Table 6:** Discriminatory accuracy of the multiple model at different cut-off of the predicted preeclampsia probability in multiparous and in primiparous women.

Centile(cut-off)	n	AR	AF	TPF (95% CI)	FPF (95% CI)	LR+ (95% CI)	LR- (95% CI)	ORa (95% CI)
***Multiparous***								
95 centile (0.07)	16847	17.5	15.9	35.5 (34.5–36.6)	4.1 (4.1–4.2)	8.58 (8.30–8.87)	0.67 (0.66–0.68)	12.8 (12.2–13.4)
97 centile (0.13)	6237	23.6	21.6	17.8 (17–18.6)	1.4 (1.4–1.5)	12.5 (11.8–13.2)	0.83 (0.83–0.84)	15 (14.1–16)
99 centile (0.25)	3160	27.5	25.3	10.5 (9.8–11.2)	0.7 (0.7–0.7)	15.4 (14.3–16.6)	0.90 (0.89–0.91)	17 (15.71–18)
***Primiparous***								
95 centile (0.12)	8884	20.8	15.7	11.7 (11.2–12.3)	2.6 (2.6–2.7)	4.46 (4.24–4.68)	0.91 (0.90–0.91)	4.92 (4.66–5.19)
97 centile (0.13)	7997	21.5	16.5	11 (10.5–11.5)	2.4 (2.3–2.4)	4.66 (4.43–4.90)	0.91 (0.91–0.92)	5.11 (4.83–5.40)
99 centile (0.19)	2392	25.6	20.2	3.9 (3.6–4.2)	0.7 (0.6–0.7)	5.85 (5.34–6.40)	0.97 (0.96–0.97)	6.04 (5.50–6.63)

AF: Attributable fraction, AR: Attributable risk, TPF: True positive fraction, ORa: mutually adjusted Odds ratios. LR+: Positive Likelihood ratio; LR-: Negative Likelihood ratio.

**Table 7:** Subgroups combinations with the highest positive likelihood ratio to rule-in preeclampsia.

Population	Multiparous	Primiparous
All	• Any combination of a risk factor with previous PE or HBP	• DM +Multiple pregnancy+ obesity • DM +HBP+ obesity • No DM + No obesity +Multiple pregnancy+ HBP • No DM + No Multiple pregnancy +Obesity + HBP
	• >97 centile of individual risk probabilities	• >99 centile of individual risk probabilities
Without Previous PE nor HBP	• Multiple pregnancy +Obese +(DM or CKD or ART or <40)	• Multiple pregnancy + Obese + Gestational diabetes
Without major risk factor	• >40 +Multiple pregnancy +ART •<40+Multiple pregnancy +ART +Obese • Multiple pregnancy + gestational diabetes +<40 +No obese + no ART	• No obese +Multiple pregnancy + ART • Obese +Multiple pregnancy +<40 +no ART

ART: assisted reproductive technologies, CKD: chronic kidney disease, DM: diabetes mellitus, HBP: chronic hypertension

## Discussion

Our population-based study confirms previous associations between PE and traditional risk factors, alone or in simple combinations. As recently recommended[23], we complement measures of associations with measure of DA. When doing so, we found that neither single risk factors alone nor their unspecific combination had an acceptable DA. Therefore, their use in clinical practice may lead to both over and under diagnoses and, thereby, unwanted consequences like over and under-treatment with LDA. However, by using *machine learning techniques* (i.e., RF) and stratification for parity, we inform the existence of individual heterogeneity in PE and identify specific combinations of specific risk factors with a high LR+ that permit a more assertive PE risk assessment for specialist referral and LDA prescription. Additionally, we demonstrate that a more extreme cut-off of the individual probabilities predicted by multiple logistic regression analysis is needed in primiparous when compared to multiparous in order to predict PE with confidence.

### Risk factors

Our results are compatible with previous findings reporting a positive association between PE and low education[17], maternal age[24], previous PE, HBP, CKD[25,26–28], ART[7], multiple pregnancy[29–31], DM and obesity [26,27,30] and a negative association with smoking before pregnancy[32]. To our knowledge this is the first study reporting an effect modification of parity by other risk factors which also justify the stratified analysis. The effect of traditional risks on the incidence of PE was lower for nulliparous than for multiparous. HBP and multiple pregnancy showed the highest negative effect on the additive scale, which highlight the importance of these risk factors mainly among multiparous. These results could be explained, at least in part, by the shorter time of exposure or less severity of diseases in nulliparous, which tend to be younger than multiparous. The interpretation of the interaction with opposite direction in the multiplicative and additive scale needs caution, and biological plausibility should be taken into consideration. For example, in the interaction between diabetes and parity, negative additive interaction seems more biologically plausible than a positive multiplicative interaction. We also found a dose response association between the number of risk factors and PE risk in multiparous, describing almost a linear trend. A non-linear effect was observed in primiparous, suggesting that the combination of clinical risk-factors among multiparous imply a more deleterious effect than in primiparous, most probably explained by the presence of previous PE only among them.

### Accuracy of high- risk groups definitions

#### Accuracy of some current definitions for high- risk groups

In everyday clinical practice the definition of high risk women varies across guidelines. In some of them[5,6] the same definition is used interchangeably for further screening tests and for prescription of LDA. However, we demonstrate that the predictive accuracy of this current practice falls to reach the standard cutoff for acceptable discrimination[7]. For example, in the whole population, primiparity vs multiparity is currently considered as a moderate risk-factor. However, our analysis indicates that its LR+ is very low (i.e., LR+ = 1.48). Besides, among primiparous, the existence of another independent risk factor did not improve PE prediction which seriously question the use of this condition alone or in unspecific bivariate combination for specialist referral or prescription of LDA. Likewise, obesity provided a LR+~ 2 and OR ~ 3 among multiparous, however in those with multiple and HBP pregnancies the presence of obesity rises the LR+ from 8 to 40. This assumption of homogeneity is one limitation of current high risk definitions for PE. That is, any single major or any combination of two moderate risk factor carries a similar PE risk and this risk is the same in multiparous and primiparous.

#### Accuracy of a new definition for high- risk groups

Applying different approaches (i.e., stratification, multiple logistic regression and random forest) we identified specific combination of risk-factors that provided a higher discriminatory accuracy to rule-in PE with confidence. For instance, among multiparous, any bivariate combination including HBP or previous PE reached a LR>10. There are few studies analyzing the impact of combinations rather than a single factor in PE risk. Interesting, despite CKD has been considered a major risk factor for PE, our results are in accordance with a previous study reporting that only those with the simultaneous presence of HBP are at a higher risk of PE[33]. We additionally identified a higher risk in those with CKD, multiple pregnancy and obesity even in the absence of HBP or previous PE. Among those without major risk factors at least three rather than two moderate factors are needed to reach a LR+>10.

In primiparous, only “rare” combinations of risk factors achieve a LR>10, particularly multiple pregnancy with HBP or with the simultaneous presence of DM and obesity. However, some combinations with a moderate LR provided an absolute risk above 30%, i.e. obesity with HBP or with DM. As observed in multiparous, the combination of multiple pregnancy and obesity was associated with an increased OR and LR+, particularly among those without major risk factors. This finding might suspect the role of volume overload in the pathogenesis of PE as a hypertensive disease[34,35]. Other factors could be needed to improve the prediction in this group. For example, a recent study in primiparous healthy women[36] has pointed out a higher risk among those with systolic blood pressure>120mmHg and maternal low birthweight, or in those with family history of PE and vaginal bleeding>5 days. We speculate that the increment in the number of risk factors might leads to an increment in preventive treatment and self-care, which could simulate the flattening in the risk of PE when three or more factors are present in primiparous.(Fig 2 and Table 6).

The contribution of traditional risk-factors to the DA for PE was modest as previously reported[37]. Using different prediction rules, the TPF varies from 18–31% for a FPR of 10% [30,38]. In our study, the AUC was significantly lower in primiparous reinforcing the necessity to identify new risk-factors in this population. These results are in contrast with a recent study reporting a similar AUC in multiparous and primiparous[39]. However, that study was performed in a higher risk population and included biomarkers, therefore results are not directly comparable. Our results agrees with Poon et al[24,40] demonstrating a better DA from multiple regression models when compared to NICE recommendations, but we additionally identify a different cut-off of the predicted probability in multiparous and primiparous to predict PE with confidence.

Despite the improvement in model fit when the subgroups were incorporated in multiple regression models, the sensitivity (TPF) and specificity (TNF) remained constant. This can be explained by the low prevalence of high-risk subgroups in the population. Contrarily to general belief, the TPF and the FPF depend of the prevalence ratio[41]. That is, the ratio between the prevalence of the risk factor and the prevalence of the disease. For the same prevalence of a disease, a risk factor with a lower prevalence (i.e., combination of risk), is related with a lower TPF and FPF when compared with a factor with a higher prevalence (i.e. single risk), since neither many cases nor many controls can be exposed to such combinations. Then, a large subset of non-exposed women develops PE possibly because of the existence of other factors that were not included in this analysis. Therefore, the DA of rare combination is generally low at the population level but could be high to predict PE at individual level.

Some researchers have pointed out that measures of association alone are unsuitable for discriminatory purpose[9,23,42]. OR is obtained by multiplying sensitivity and specificity (TP*TN/FN*FP). Then, the same OR can be obtained with very different scenarios of sensitivity and specificity. Therefore, the traditional OR approach prevent a more personalized medicine. Therefore, we propose the use of measures of DA to disentangle the utility of a risk-factor for screening or treatment purposes. The main advantage of LR versus sensitivity and specificity is that clinicians can use them to quantify the probability of a disease for an individual patient. The LR summarizes how many times more (or less) likely patients with the PE are to have that particular risk factor (or combination) than patients without the disease[21,43].

### Strength and limitations

To our knowledge, this is the first study explicitly focused on understanding heterogeneity in PE risk in order to identify high-risk subgroups of PE by combining specific risk-factors. While most previous studies have provided risk equations for the whole population of women[24,44] or mainly focused in primiparous[36], we stratified the analysis by parity. We included all pregnancies even in the presence of congenital malformation (ICD-10 codes Q00-Q99) or HBP to extend the results in real clinical settings. Additionally, we included post-partum PE that is usually excluded when data is exclusively based on birth registers. As there are many possible combinations of variables, we used a machine learning approach by RF algorithm as a guide to identify the most important variables for subsequently generate subgroups at a higher-risk of PE.

This study also has potential limitations. First, we have excluded missing data, but we cannot assure that missing values were completely at random even if included and excluded deliveries were balanced concerning the prevalence important risk-factors. Second, we had no information on interventions during pregnancy, such as aspirin prophylaxis. At the study interval, there was no preeclampsia screening in place, however, some patients at risk were possibly on LDA treatment, particularly women with prior preterm preeclampsia, i.e., probably around 2%-5% of all preeclampsia cases, which might bias the estimations towards the null in this population. Third, we were not able to evaluate all possible combinations of moderate risk-factors reported in published guidelines, neither include the histories of previous PE from longer pregnancy intervals. Fourth, we have not validated our finding in a separate population or by mean of bootstraps so we cannot rule out that our multiple regression model might be overfitted and the AUCs overestimated. This is particularly important with the results from the smaller subgroups that may be the product of overfitting and may not readily reproduce in other study samples. We have used dichotomous variables in other to adopt a similar approach as that used in current guidelines, however ordinal or continuous variables could produce more precise estimations. Finally, even though the quality of the MBR seems appropriate[15], our results need to be validated in other populations.

## Conclusions

No one risk-factors alone or unspecific combinations reached an acceptable accuracy, and ≥3 moderate risk combinations are needed in those without major risk-factors to reach a LR+> 10. Consequently, current approach based exclusively in OR, might be associated with inefficient specialist referral and unnecessary treatment with LDA. The prediction of PE was improved with a more individualized approach, by identifying specific combinations or by defining a differential cut-off to the distribution of the predicted probability for multiparous and nulliparous obtained by multiple regression analysis. However, the absence of any single neither relevant combinations were enough to rule-out the disease. The identification of such specific subgroups can improve the reliability of LDA prescription, but those with any single risk might need further screening. Our results contribute to a more personalized risk estimation of preeclampsia.

## Supporting information

S1 Table Characteristics of included and missing data among multiparous and primiparous women. (DOCX)

S2 Table Bivariate interaction effect between parity and each risk factors in multiple logistic regression models. One interaction term was included in each model. Previous preeclampsia was considered a dummy variable with three categories: yes or no in multiparous and no applicable in primiparous.
(DOCX)

S3 Table Association between number of clinical risk factor and the risk of PE in both primiparous and multiparous adjusted by smoking, education and family situation. (DOCX)

S4 Table Discriminatory accuracy of specific bivariate combinations of risk factor for preeclampsia in the overall population of pregnancies without major risk factors. (DOCX)

S5 Table Discriminatory accuracy of specific bivariate combinations of risk factor for preeclampsia in multiparous and primiparous pregnancies without major risk factors. (DOCX)
