# Supplementary material for: Revisiting the discriminatory accuracy of traditional risk factors in preeclampsia screening
Source: PLoS One. 2017 May 25;12(5):e0178528. doi: 10.1371/journal.pone.0178528 (PMC5444844; doi:10.1371/journal.pone.0178528)
Supplement: S1 Table — (DOCX) [file pone.0178528.s001.docx]

**S1Table. Characteristics of included and missing data among multiparous and primiparous women**

|  | Multiparous | | Primiparous | |
| --- | --- | --- | --- | --- |
|  | Included | Missing | Included | Missing |
| N | 344001 | 47007 | 282599 | 45454 |
| Preeclampsia, % | 2.41 | 2.64 | 5.57 | 5.60 |
| Maternal age>40., % | 4.54 | 5.46 | 1.62 | 1.82 |
| HBP, % | 0.83 | 0.78 | 0.73 | 0.60 |
| DM, % | 0.73 | 0.57 | 0.60 | 0.40 |
| CKD, % | 0.54 | 0.38 | 0.47 | 0.34 |
| Autoimmune, % | 0.22 | 0.21 | 0.22 | 0.17 |
| Multiple pregnancy, % | 1.64 | 1.91 | 1.22 | 1.52 |
| Previous PE in parous, % | 3.90 | 3.73 | - | - |
| ART | 1.50 | 0.90 | 3.93 | 2.01 |
| Gestational diabetes | 0.86 | 0.89 | 0.73 | 0.75 |

ART: assisted reproductive technology, CKD: Chronic kidney disease, DM: Diabetes mellitus, HBP: chronic hypertension
