# Supplementary material for: Revisiting the discriminatory accuracy of traditional risk factors in preeclampsia screening
Source: PLoS One. 2017 May 25;12(5):e0178528. doi: 10.1371/journal.pone.0178528 (PMC5444844; doi:10.1371/journal.pone.0178528)
Supplement: S2 Table — One interaction term was included in each model. Previous preeclampsia was considered a dummy variable with three categories: yes or no in multiparous and no applicable in primiparous. (DOCX) [file pone.0178528.s002.docx]

**S2 Table. Bivariate interaction effect between parity and each risk factors in multiple logistic regression models. Previous preeclampsia was considered a dummy variable with three categories: yes or no in multiparous and no applicable in primiparous.**

| Risk factor | Ratio of ORs (CI95%) | Interaction contrast ratio (CI95%) |
| --- | --- | --- |
| Maternal age>40 | 0.81(0.70-0.93) | -0.62(-0.78- -0.45) |
| Low Educational status | 0.93(0.88-0.99) | -.016(-0.21- -0.10) |
| Family situation | 0.73(0.64-0.84) | -0.29(-0.45- -0.13) |
| DM | 1.55(1.26-1.90) | -0.62(-1.08- -0.15) |
| HBP | 0.87(0.74-1.04) | -2.52(-3.19- -1.97) |
| Autoimmune | 0.70(0.42-1.16) | -0.53(-1.13-0.05) |
| CKD | 0.62(0.46-0.84) | -1.04(-1.52- -0.55) |
| Obesity | 1.03(0.97-1.10) | -0.92(-1.05- -0.79) |
| Smoke | 0.90(0.83-0.97) | 0.51(0.48-0.54) |
| Multiple Pregnancy | 0.74(0.65-0.85) | -3.52(-4.08- -2.95) |
| ART | 0.65(0.55-0.66)* | -0.72(-0.96- -0.47) |
| Gestational Diabetes | 1.08(0.87-1.33) | -0.52(-0.84- -0.19) |

ART: assisted reproductive technology, CKD: Chronic kidney disease, DM: Diabetes mellitus, HBP: chronic hypertension
