# Supplementary material for: Revisiting the discriminatory accuracy of traditional risk factors in preeclampsia screening
Source: PLoS One. 2017 May 25;12(5):e0178528. doi: 10.1371/journal.pone.0178528 (PMC5444844; doi:10.1371/journal.pone.0178528)
Supplement: S3 Table — (DOCX) [file pone.0178528.s003.docx]

**S3 Table. Association between number of clinical risk factor and the risk of PE in both primiparous and multiparous adjusted by smoking, education and family situation**

|  | Multiparous | | | | | | Primiparous | | |
| --- | --- | --- | --- | --- | --- | --- | --- | --- | --- |
|  | Overall | | | Without previous_PE | | |  |  |  |
| Number of Risk factors | N | AR | ORa (95% CI) | N | AR | ORa (95% CI) | N | AR | ORa (95% CI) |
| 0 | 262839 | 1.2 | Ref | 262389 | 1.12 | Ref | 233675 | 4.36 | Ref |
| 1 | 69137 | 4.93 | 4.55(4.33-4.79) | 60317 | 3.57 | 3.19(3.04-3.35) | 43517 | 10.4 | 2.51(2.48-2.61) |
| 2 | 10477 | 15.0 | 15.5(14.5-16.5) | 6689 | 9.37 | 8.86(8.09-9.70) | 4796 | 18.9 | 5.03(4.67-5.42) |
| 3 | 1370 | 23.3 | 26.4(23.1-30.1) | 667 | 15.1 | 15.3(12.3-18.3) | 535 | 20.6 | 5.56(4.50-6.87) |
| ≥4 | 178 | 28.1 | 33.8(24.3-46.9) | 82 | 14.6 | 14.9(8.03-27.5) | 76 | 17.1 | 4.44(2.44-8.08) |
| AUC |  |  | 73.6(73-74.2) |  |  | 67.1(66.3-67.8) |  |  | 61.1(60.6-61.6) |
| Sensitivity (FP10%) |  |  | 24.2(23.3-25.1) |  |  | 14.2(13.3-15) |  |  | 20.1(19.5-20.8) |
| AIC |  |  | 69959 |  |  | 55577 |  |  | 117936 |

Values are: AIC: Akaike information criteria, AR: Attributable risk
