# Supplementary material for: Revisiting the discriminatory accuracy of traditional risk factors in preeclampsia screening
Source: PLoS One. 2017 May 25;12(5):e0178528. doi: 10.1371/journal.pone.0178528 (PMC5444844; doi:10.1371/journal.pone.0178528)
Supplement: S4 Table — (DOCX) [file pone.0178528.s004.docx]

**S4 Table. Discriminatory accuracy of specific bivariate combinations of risk factor for preeclampsia in the overall population of pregnancies without major risk factors.**

| Combination | n | AR | AF | TPF | FPF | LR+ | LR- | ORa |
| --- | --- | --- | --- | --- | --- | --- | --- | --- |
| None | 552541 | 2.72 | Ref | | | | | |
| Obesity+Multiple | 592 | 11.2 | 8.43 | 0.3  (0.3-0.3) | 0.1  (0.1-0.1) | 3.65  (2.83-4.71) | ̴ 1 | 4.43  (3.42-5.73) |
| Obesity+ART | 381 | 6.56 | 3.84 | 0.1  (0.1-0.2) | 0.1  (0.1-0.1) | 2.04  (1.36-3.06) | ̴ 1 | 2.47  (1.65-3.72) |
| Obesity+Gestational diabetes | 893 | 8.40 | 5.68 | 0.4  (0.3-0.5) | 0.1  (0.1-0.1) | 2.67  2.11-3.38) | ̴ 1 | 3.22  (2.54-4.08) |
| Obesity+Maternal age | 1647 | 6.62 | 3.9 | 0.5  (0.4-0.7) | 0.3  (0.3-0.3) | 2.06  (1.70-2.50) | ̴ 1 | 2.48  (2.03-3.01) |
| Obesity+Primiparity | 24713 | 11 | 8.27 | 13.6  (13.1-14.1) | 0.8  (0.7-3.8) | 3.59  (3.46-3.73) | 0.90  (0.89-0.90) | 4.37  (4.18-4.56) |
| Muliple+ART | 546 | 13.9 | 11.2 | 0.4  (0.3-05) | 0.1  (0.1-0.1) | 4.71  (3.69-5.99) | ̴ 1 | 5.77  (4.52-7.37) |
| Multiple+Gestational diabetes | 37 | 10.8 | 8.09 | ̴ 0 | ̴ 0 | 3.53  (1.25-9.96) | ̴ 1 | 4.25  (1.50-12.03) |
| Multiple+Maternal age | 226 | 11.9 | 9.23 | 0.1  (0.1-0.1) | ̴ 0 | 3.95  (2.64-5.90) | ̴ 1 | 4.79  (3.21-7.16) |
| Multiple+Primiparity | 2611 | 18.6 | 15.9 | 2.4  (2.2-2.7) | 0.4  0.4-0.4) | **6.66**  **(6.04-7.34)** | 0.98  (0.98-0.98) | 8.23  (7.45-9.10) |
| ART+Gestational diabetes | 39 | 10.3 | 7.54 | ̴ 0 | ̴ 0 | 3.33  (1.18-9.36) | ̴ 1 | 4  (1.42-11.26) |
| ART+Maternal age | 649 | 5.70 | 2.98 | 0.2  (0.1-0.3) | 0.1  (0.1-0.1) | 1.76  (1.26-2.45) | ̴ 1 | 2.16  (1.55-3.02) |
| ART+Primiparity | 9906 | 7.04 | 4.32 | 3.5  (3.2-3.8) | 1.6  (1.6-1.6) | 2.20  (2.04-2.38) | 0.98  (0.98-0.98) | 2.73  (2.52-2.95) |
| Gestational diabetes+Maternal age | 248 | 7.66 | 4.94 | 0.1  (0.1-0.1) | ̴ 0 | 2.41  (1.51-3.85) | ̴ 1 | 2.90  (1.81-4.63) |
| Gestational diabetes+Primiparity | 1917 | 13.3 | 10.5 | 1.3  (1.4-1.4) | 0.3  (03-0.3) | 4.44  (3.90-5.07) | 0.99  (0.99-0.99) | 5.44  (4.76-6.21) |
| Maternal age+Primiparity | 4399 | 8.62 | 5.9 | 1.6  (1.7-2.1) | 0.7  (0.7-.7) | 2.74  (2.47-3.05) | 0.99  (0.99-0.99) | 3.33  (2.99-3.71) |

ART: Assistive reproductive technologies
