# Supplementary material for: Revisiting the discriminatory accuracy of traditional risk factors in preeclampsia screening
Source: PLoS One. 2017 May 25;12(5):e0178528. doi: 10.1371/journal.pone.0178528 (PMC5444844; doi:10.1371/journal.pone.0178528)
Supplement: S5 Table — (DOCX) [file pone.0178528.s005.docx]

**S5 Table. Discriminatory accuracy of specific bivariate combinations of risk factor for preeclampsia in multiparous and primiparous pregnancies without major risk factors**

| Combination | | | | n | AR | AF | TPF | FPF | LR+ | LR- | ORa |
| --- | --- | --- | --- | --- | --- | --- | --- | --- | --- | --- | --- |
| ***Multiparous*** | | | | | | | | | | | |
| Multiple | Obese | >40 | ART | 264215 | 1.13 | Reference | | | | | |
| 0 | 0 | 0 | 1 | 3284 | 2.04 | 0.91 | 1.3  (1-1.6) | ̴ 1 | 1.26  (0.99-1.61) | 1  (0.99-1) | 1.85  (1.44-2.36) |
| 0 | 0 | 1 | 0 | 11991 | 2.24 | 1.11 | 5.1  (4.5-5.7) | 3.7  (3.6-3.7) | 1.39  (1.24-1.57) | 0.99  (0.98-0.99) | 1.99  (1.75-2.26) |
| 0 | 0 | 1 | 1 | 512 | 3.32 | 2.19 | 0.3  (0.2-0.5) | 0.2  (0.1-0.2) | 2.08  (1.29-3.38) | ̴ 1 | 3.03  (1.86-4.92) |
| 0 | 1 | 0 | 0 | 36569 | 3.53 | 2.4 | 24.6  (23.4-25.8) | 11.1  (11-11.1) | 2.22  (2.12-2.33) | 0.85  (0.84-0.86) | 3.03  (2.89-3.31) |
| 0 | 1 | 0 | 1 | 394 | 6.60 | 5.47 | 0.5  (0.3-0.7) | 0.1  (0.10.1) | 4.29  (2.88-6.37) | ̴ 1 | 6.02  (4.04-8.99) |
| 0 | 1 | 1 | 0 | 1746 | 6.76 | 5.63 | 2.2  (3.65-5.29) | 0.5  (0.5-0.5) | 4.40  (3.65-5.29) | 0.98  (0.98-0.98) | 6.06  (5-7.33) |
| 0 | 1 | 1 | 1 | 72 | 8.33 | 7.2 | 0.1(0-0.2) | ̴ 0 | 5.51  (2.39-12.7) | ̴ 1 | 7.86  (3.71-18.1) |
| 1 | 0 | 0 | 0 | 3875 | 7.77 | 6.64 | 5.7  (5.1-6.4) | 1.1  (1.1-1.2) | 5.11  (4.59-5.73) | 0.95  (0.95-.96) | 7.40  (6.54-8.38) |
| 1 | 0 | 0 | 1 | 505 | 11.88 | 10.8 | 1.1  (0.9-1.5) | 0.1  (0.1-0.2) | 8.18  (625-10.7) | 0.99  (0.99-0.99) | 11.9  (9.02-34.9) |
| 1 | 0 | 1 | 0 | 202 | 12.4 | 11.1 | 0.5  (0.3-0.7) | 0.1  (0-0.1) | 8.57  (5.64-13.01) | ̴ 1 | 12.3  (8.08-18.6) |
| 1 | 0 | 1 | 1 | 65 | 16.9 | 15.8 | 0.2  (0.1-0.4) | ̴ 0 | **12.4**  **(6.46-23.6)** | ̴ 1 | 18.5  (9.64-35) |
| 1 | 1 | 0 | 0 | 609 | 10.8 | 9.71 | 1.3  (1-1.6) | 0.2  (0.2-0.2) | 7.37(5.72-9.50) | 0.99  (0.99-0.99) | 10.3  (7.98-13.4) |
| 1 | 1 | 0 | 1 | 46 | 34.8 | 33.7 | 0.3  (0.2-0.5) | ̴ 0 | **32.4**  **(17.7-59.3)** | ̴ 1 | 11.6  (24.2-82.2) |
| 1 | 1 | 1 | 0 | 27 | 11.1 | 9.98 | 0.1  (0-0.2) | ̴ 0 | 7.58  (2.28-25.2) | ̴ 1 | 9.81  (2.92-32.9) |
| 1 | 1 | 1 | 1 | 12 | 33.3 | 32.2 | 0.1  (0-0.2) | ̴ 0 | **30.3**  **(9.14-100)** | ̴ 1 | 46.2  (24.2-82.1) |
| **AUC 65.19(64.39-65.99) Sensitivity (FP10%) 27.98(26.8-29.8) AIC:51449** | | | | | | | | | | | |
| ***Primiparous*** | | | | | | | | | | | |
| Multiple | Obese | >40 | ART | 234848 | 4.38 | Reference | | | | | |
| 0 | 0 | 0 | 1 | 8440 | 5.23 | 0.85 | 3  (2.7-3.3) | 3  (3-4.1) | 098  (0.89-1.08) | ̴ 1 | 1.18(1.07-1.30) |
| 0 | 0 | 1 | 0 | 3124 | 7.30 | 2.92 | 1.5  (1.4-1.8) | 1.1  (1.1-1.1) | 1.40  (1.23-1.60) | 1  (0.99-1) | 1.71(1.49-1.96) |
| 0 | 0 | 1 | 1 | 650 | 6.77 | 2.39 | 0.3  (0.2-0.4) | 0.2  (02-0.2) | 1.29  (0.95-1.76) | ̴ 1 | 1.56(1.14-2.12) |
| 0 | 1 | 0 | 0 | 25335 | 11.3 | 6.88 | 19.4  (18.7-20) | 8.6  (0.5-8.7) | 2.26  (2.18-2.34) | 0.88  (0.87-0.89) | 2.75(2.63-3.98) |
| 0 | 1 | 0 | 1 | 935 | 13.5 | 9.1 | 0.9  (0.7-1) | 0.3  (0.3-0.3) | 2.78  (2.30-3.35) | 0.99  (0.99-1) | 3.30(2.73-3.98) |
| 0 | 1 | 1 | 0 | 405 | 16.1 | 11.7 | 0.4  (0.3-0.6) | 0.1  (0.1-0.1) | 3.41  (2.62-4.44) | ̴ 1 | 4.11(3.15-5.36) |
| 0 | 1 | 1 | 1 | 93 | 13.9 | 9.6 | 0.1  (0-0.2) | ̴ 0 | 2.90  (1.61-5.21) | ̴ 1 | 3.47(1.93-6.24) |
| 1 | 0 | 0 | 0 | 2356 | 17.8 | 13.5 | 2.9  (2.6-3.1) | 0.7  (0.7-0.8) | 3.87  (3.49-4.29) | 0.98  (0.98-0.98) | 4.74(4.26-5.28) |
| 1 | 0 | 0 | 1 | 569 | **23.2** | 18.8 | 0.9  (0.8-1.1) | 0.2  (0.2-0.2) | **5.39**  **(4.44-6.54)** | 0.99  (0.99-0.99) | 6.45(5.30-7.85) |
| 1 | 0 | 1 | 0 | 52 | **28.9** | 24.5 | 0.1  (0.1-0.2) | ̴ 0 | **7.23**  **(3.97-13.2)** | 1 | 8.76(4.82-15.9) |
| 1 | 0 | 1 | 1 | 60 | 20 | 15.6 | 0.1  (0-0.1) | ̴ 0 | 4.46(2.37-8.39) | 1 | 5.36(2.83-10.2) |
| 1 | 1 | 0 | 0 | 279 | **26.2** | 21.8 | 0.5  (0.4-0.6) | 0.1  (0.1-0.1) | **6.32**  **(4.84-8.25)** | 1 | 7.65(5.85-10) |
| 1 | 1 | 0 | 1 | 60 | 13.3 | 8.92 | 0.1  (0-0.1) | ̴ 0 | 2.74  (1.30-5.77) | 1 | 3.25(1.54-6.84) |
| 1 | 1 | 1 | 0 | 5 | 20 | 15.6 | ̴ 0 | ̴ 0 | 4.46  (0.50-39) | 1 | 5.34(0.58-48.9) |
| 1 | 1 | 1 | 1 | 10 | 10 | 5.62 | ̴ 0 | ̴ 0 | 1.98  (0.25-15.6) | 1 | 2.29(0.29-17.9) |
| **AUC 59.64(59.14-60.13) Sensitivity (FP10%) 24.5 (23.6-25.5) AIC:112365** | | | | | | | | | | | |

Values are: AIC: Akaike information criteria, AF: Attributable fraction, AR: Attributable risk, TPF: True positive fraction, ORa: mutually adjusted Odds ratios. LR+: Positive Likelihood ratio; LR-: Negative Likelihood ratio.

ART: Assistive reproductive technologies
